# Value of Contrast-Enhanced Ultrasound in Adjusting the Classification of Chinese-TIRADS 4 Nodules

**DOI:** 10.1155/2022/5623919

**Published:** 2022-01-06

**Authors:** Hong Cheng, Shuang-Shuang Zhuo, Xin Rong, Ting-Yue Qi, Hong-Guang Sun, Xiao Xiao, Wen Zhang, Hai-Yan Cao, Lin-Hai Zhu, Lei Wang

**Affiliations:** ^1^Department of Ultrasound, Hangzhou Fuyang District First People's Hospital, Hangzhou, Zhejiang 311400, China; ^2^Department of Ultrasound, Medical Imaging Center, Affiliated Hospital of Yangzhou University, Yangzhou University, Yangzhou 225012, China; ^3^Department of Thyroid and Breast Surgery, Affiliated Hospital of Yangzhou University, Yangzhou University, Yangzhou 225012, China; ^4^Department of Pathology, Affiliated Hospital of Yangzhou University, Yangzhou University, Yangzhou 225012, China

## Abstract

**Objectives:**

To explore the value of applying contrast-enhanced ultrasound (CEUS) in adjusting the classification of category 4 nodules in the Chinese-Thyroid Imaging Report and Data System (C-TIRADS).

**Methods:**

The data of preoperative conventional ultrasound and CEUS examinations of 125 C-TIRADS 4 nodules in 109 patients were retrospectively analyzed. We divided the thyroid nodules into two groups based on whether recommend by the guide fine-needle aspiration (FNA). Group I included C-TIRADS 4A nodules with a maximum diameter ≤15 mm and C-TIRADS 4B and 4C nodules with a maximum diameter ≤10 mm, and Group II included C-TIRADS 4A nodules with a maximum diameter >15 mm and C-TIRADS 4B and 4C nodules with a maximum diameter >10 mm. In CEUS, thyroid nodules showing suspicious malignant features such as hypoenhancement or early washout were adjusted to a level higher in the C-TIRADS classification; thyroid nodules showing possible benign features such as iso- or hyperenhancement were adjusted to a level lower; and thyroid nodules showing no enhancement were adjusted to C-TIRADS 3. Taking the pathological results as the gold standard, the receiver operating characteristic (ROC) curves of the C-TIRADS classification before and after the adjustment based on CEUS were plotted, and the diagnostic efficiency was compared.

**Results:**

The sensitivity, specificity, accuracy, and positive and negative predictive values of the C-TIRADS classification for the diagnosis of thyroid nodule malignancy before the adjustment based on the CEUS results were 83.6%, 63.8%, 74.4%, 72.7%, and 77.1%, respectively, and these values were 91.0%, 82.8%, 87.2%, 85.9%, and 88.9%, respectively, after the adjustment. The area under the ROC curve (AUC) was 0.737 and 0.869, respectively, showing a significant difference (*Z* = 3.288, *P*=0.001). The diagnostic efficiency of C-TIRADS classification after the adjustment based on the CEUS results in both groups was improved compared with the result before the adjustment, and the difference in Group II was significant (*Z* = 2.931, *P*=0.003).

**Conclusions:**

CEUS significantly improved the diagnostic performance in the adjustment of C-TIRADS 4 nodule classification, especially for the nodules which needs FNA recommended by the C-TIRADS.

## 1. Introduction

The incidence of thyroid cancer has steadily increased in China in recent years [1]. Ultrasound examination is the most important imaging method to assess the malignant risk of thyroid nodules [2]. The Chinese-Thyroid Imaging Report and Data System (C-TIRADS) guidelines were formulated by the Superficial Organ and Vascular Ultrasound Group of the Society of Ultrasound in Medicine of the Chinese Medical Association in 2020. Due to overlapping in the imaging features of benign and malignant thyroid nodules in conventional ultrasound, the malignant risk of category 4 nodules in C-TIRADS (i.e., C-TIRADS 4 nodules) ranges from 2% to 90%. Contrast-enhanced ultrasound (CEUS) is a new technique that reflects the microvascular conditions inside tumors. However, few studies on CEUS examination of C-TIRADS 4 nodules are available. This study explored the application value of CEUS in adjusting the classification of C-TIRADS 4 nodules of different sizes.

## 2. Materials and Methods

### 2.1. Study Subjects

This study retrospectively analyzed the clinical data of 125 thyroid nodules of 109 patients from January 2016 to December 2020 in our institution, with all patients meeting the inclusion criteria as follows: (1) underwent surgery and had pathological results; (2) had C-TIRADS 4 nodules and underwent conventional ultrasound and CEUS examinations before surgery; (3) had the maximum nodular diameter of 5–30 mm; and (4) had solid or mostly solid structures in the thyroid nodules. The exclusion criteria of this study were as follows: (1) mainly cystic structures in the thyroid nodules; (2) maximum nodular diameter >30 mm or <5 mm; (3) nodule location too deep; and (4) incomplete imaging data. The thyroid nodules were divided into two groups based on whether the C-TIRADS guidelines recommend fine-needle aspiration (FNA) [3]: Group I included C-TIRADS 4A nodules with a maximum diameter ≤15 mm and C-TIRADS 4B and 4C nodules with a maximum diameter ≤10 mm and Group II included C-TIRADS 4A nodules with a maximum diameter >15 mm and C-TIRADS 4B and 4C nodules with a maximum diameter >10 mm. The study was approved by our local ethics committee (approval number: 2020-YKL014-014), and written informed consent was obtained from all patients.

### 2.2. Instruments and Examination Methods

The MyLab Twice color Doppler ultrasound scanner (Esaote, Italy) was used in this study. The scanner was equipped with CnTi imaging software and a model LA523 high-frequency linear transducer array. The contrast agent used for CEUS was SonoVue (Bracco, Italy). During the examinations, the patients were in a supine position to fully expose the examination site. For conventional ultrasound examination, the maximum nodular diameter was measured, imaging features of the thyroid nodules were recorded, and static and continuous dynamic ultrasound images were stored. The planes showing the complete nodule and sufficient surrounding normal thyroid tissue were selected for CEUS imaging, using a dual-scan mode, where the CEUS image is shown on the right side and the gray-scale image is shown on the left side of the screen simultaneously. SonoVue was prepared in a standard fashion, and 2 ml was administered as a bolus injection through a 20-gauge i.v. cannula introduced into the cubital vein, followed by a flush with 5 ml of 0.9% normal saline solution. The entire dynamic process of radiography was stored in real time. For patients with multiple nodules, imaging was repeated 15 minutes after the first imaging.

### 2.3. Image Analysis

Two attending radiologists who had more than two years of experience with thyroid CEUS examination independently analyzed the stored imaging data to score the thyroid nodules in accordance with the C-TIRADS guidelines and assessed the classification of the nodules. Results where the two radiologists were in agreement were the final diagnostic results, and results without an agreement were further investigated by a third radiologist to determine the final diagnosis. Thyroid nodules with score 1 were classified as C-TIRADS 4A nodules; thyroid nodules with score 2 were classified as C-TIRADS 4B nodules; and thyroid nodules with scores 3-4 were classified as C-TIRADS 4C nodules [3]. The classification of C-TIRADS was adjusted according to the CEUS imaging features of the nodules: thyroid nodules showing suspicious malignant features, such as hypoenhancement or early washout, were adjusted to a level higher in the C-TIRADS classification; thyroid nodules showing possible benign features, such as iso- or hyperenhancement, were adjusted to a level lower in the C-TIRADS classification; and thyroid nodules showing no enhancement or sparse spot or line enhancement were adjusted to C-TIRADS 3 [3]. Thyroid nodules with both benign and malignant signs had their C-TIRADS classification adjusted according to the malignancy. In this study, nodules with C-TIRADS 4A and below were classified as benign thyroid nodules, whereas nodules with C-TIRADS 4B and above were classified as malignant thyroid nodules.

### 2.4. Statistical Analysis

MedCalc 15.8 software was used for statistical analysis of the data. The measurement data were presented as mean ± standard deviation ($\bar{x}$ ± SD), and the comparison of the mean was performed using an independent sample *t*-test. Comparison of the proportions was performed using Pearson's chi-square test. Taking the pathological results as the reference, the sensitivity, specificity, accuracy, positive predictive value, and negative predictive value of C-TIRADS before and after the adjustment based on CEUS for the diagnosis of thyroid nodule malignancy were calculated. Subsequently, the receiver operating characteristic (ROC) curve was plotted. *P* < 0.05 was considered to indicate a statistically significant difference.

## 3. Results

### 3.1. Pathological Results

In this study, there were 109 patients (35 males and 74 females) ranging in age from 27 to 77 years, with a mean age of 47.32 ± 12.51 years. Among the 125 thyroid nodules of these patients, the maximum nodular diameter ranged from 5 to 30 mm, and the average maximum nodular diameter was 13.15 ± 5.87 mm.

According to the pathological results, there were 67 malignant thyroid nodules, of which 91.1% (61/67) were papillary thyroid carcinomas (PTCs), including 29 papillary thyroid microcarcinomas (PTMCs). Among all malignant thyroid nodules, there were 6.0% (4/67) follicular thyroid cancer and 2.9% (2/67) medullary thyroid carcinoma. This study also included 58 benign thyroid nodules. Among them, 58.6% (34/58) were nodular goiter, 27.6% (16/58) were follicular adenomas, 6.9% (4/58) were subacute granulomatous thyroiditis, and 6.9% (4/58) were chronic lymphocytic thyroiditis.

### 3.2. Adjustment of C-TIRADS 4 Nodules Based on the Results of CEUS Examination

**Figure 1:**
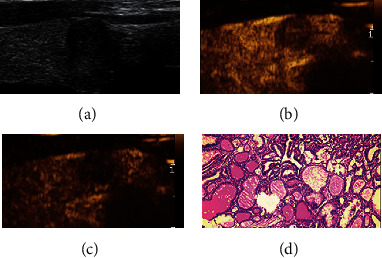
Adjustment of C-TIRADS classification of thyroid nodule with hypoenhancement in CEUS. A 57-year-old female patient with a right thyroid nodule, maximum diameter of 10 mm, which was originally classified as C-TIRADS 4A before the adjustment based on the CEUS results. The nodule was reclassified as C-TIRADS 4B after the adjustment. (a) Two-dimensional gray scale. (b) Hypoenhancement at 17 s in CEUS. (c) Very hypoenhancement at 37 s in CEUS. (d) Diagnosed as papillary thyroid carcinoma in histopathology (HE × 100). CEUS: contrast-enhanced ultrasound; C-TIRADS: the Chinese-Thyroid Imaging Report and Data System.

**Figure 2:**
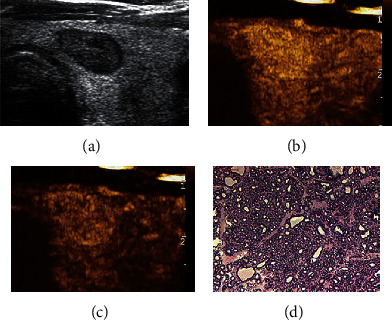
Adjustment of C-TIRADS classification of thyroid nodule with hyperenhancement in CEUS. A 61-year-old male patient with a left thyroid nodule, maximum diameter of 16 mm, which was originally classified as C-TIRADS 4A before the adjustment based on the CEUS results. The nodule was reclassified as C-TIRADS 3 after the adjustment. (a) Two-dimensional gray scale. (b) Hyperenhancement at 17 s in CEUS. (c) Still hyperenhancement at 37 s in CEUS. (d) Diagnosed as thyroid follicular adenoma (HE × 100). CEUS: contrast-enhanced ultrasound; C-TIRADS: the Chinese-Thyroid Imaging Report and Data System.

**Figure 3:**
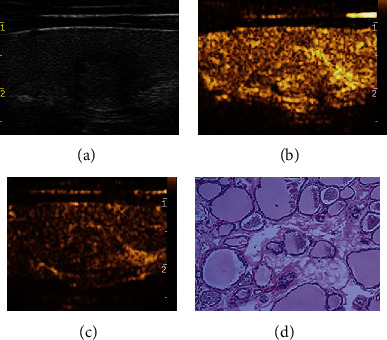
Adjustment of C-TIRADS classification of thyroid nodule with isoenhancement in CEUS. A 60-year-old female patient with a left thyroid nodule, maximum diameter of 11 mm, which was originally classified as C-TIRADS 4B before the adjustment based on the CEUS results. The nodule was reclassified as C-TIRADS 4A after the adjustment. (a) Two-dimensional gray scale. (b) Isoenhancement at 17 s in CEUS. (c) Isoenhancement at 37 s in CEUS. (d) Diagnosed as nodular goiter in histopathology (HE × 100). CEUS: contrast-enhanced ultrasound; C-TIRADS: the Chinese-Thyroid Imaging Report and Data System.

**Figure 4:**
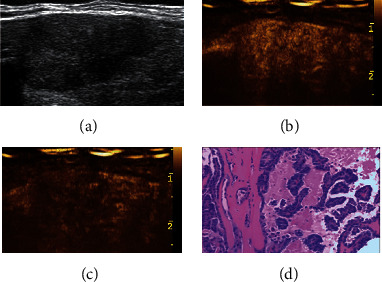
Adjustment of C-TIRADS classification of a thyroid nodule with early washout in CEUS. A 37-year-old male patient with a right thyroid nodule, maximum diameter of 23 mm, which was originally classified as C-TIRADS 4A before the adjustment based on the CEUS results. The nodule was reclassified as C-TIRADS 4B after the adjustment. (a) Two-dimensional gray scale. (b) Hyperenhancement at 15 s in CEUS. (c) Hypoenhancement with early washout at 21 s in CEUS. (d) Diagnosed as papillary thyroid carcinoma in histopathology (HE × 100). CEUS: contrast-enhanced ultrasound; C-TIRADS: the Chinese-Thyroid Imaging Report and Data System.

**Figure 5:**
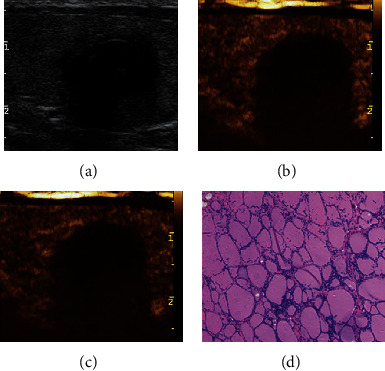
Adjustment of C-TIRADS classification of thyroid nodule with no enhancement in CEUS. A 63-year-old male patient with a right thyroid nodule, maximum diameter of 20 mm, which was originally classified as C-TIRADS 4C before the adjustment based on the CEUS results. The nodule was reclassified as C-TIRADS 3 after the adjustment. (a) Two-dimensional gray scale. (b) No enhancement at 17 s in CEUS. (c) No enhancement at 37 s in CEUS. (d) Diagnosed as nodular goiter combined with collagen degeneration and focal necrosis (HE × 100). CEUS: contrast-enhanced ultrasound; C-TIRADS: the Chinese-Thyroid Imaging Report and Data System.

**Figure 6:**
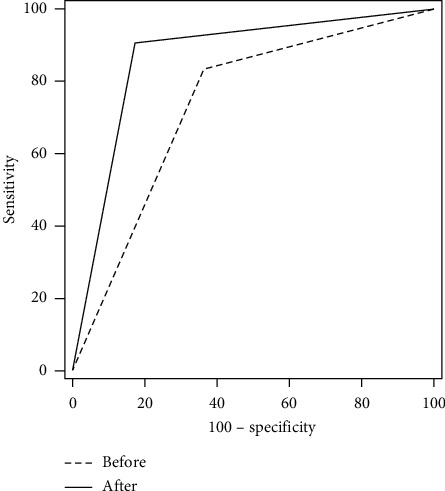
Receiver operating characteristic curves before and after the adjustment of C-TIRADS classification based on the CEUS results. CEUS: contrast-enhanced ultrasound; C-TIRADS: the Chinese-Thyroid Imaging Report and Data System.

The conventional ultrasound diagnosis of 125 nodules were C-TIRADS 4A, 4B, and 4C at 38.4% (48 / 125), 30.4% (38 / 125), and 31.2% (39 / 125), respectively. According to the CEUS results, 53 showed hypoenhancement, 27 showed isoenhancement, 32 showed hyperenhancement, 13 showed no enhancement, and 38 showed early washout. After the adjustment based on CEUS results, the thyroid nodules were classified as C-TIRADS 3, C-TIRADS 4A, C-TIRADS 4B, C-TIRADS 4C, and C-TIRADS 5, accounting for 38.4% (48/125), 4.8% (6/125), 19.2% (24/125), 20.8% (26/125), and 16.8% (21/125) of the total nodules, respectively (Figures 1–5). Using C-TIRADS 4A as the cutoff, the diagnostic efficiency in distinguishing benign and malignant thyroid nodules after the adjustment based on the CEUS results was significantly higher than before the adjustment, with significant differences in the area under the ROC curves (AUCs) (0.869 vs. 0.737, *P*=0.001) (Table 1 and Figure 6).

### 3.3. Results of C-TIRADS Classification Adjustment Based on CEUS Results for Thyroid Nodules of Different Sizes

Table 1 shows the results of adjustment of the C-TIRADS classification based on the CEUS results for thyroid nodules of different sizes. In Group I, there were 55 thyroid nodules, with the percentages of C-TIRADS 4A, C-TIRADS 4B, and C-TIRADS 4C of 40.0% (22/55), 25.5% (14/55), and 34.5% (19/55), respectively. Using C-TIRADS 4A as the cutoff, the diagnostic performance after the adjustment based on CEUS results was improved compared with that before the adjustment, but the AUCs had no significant difference (0.802 vs. 0.733, *P* > 0.05).

Among the 70 thyroid nodules in Group II, the percentages of C-TIRADS 4A, C-TIRADS 4B, and C-TIRADS 4C were 37.1% (26/70), 34.3% (24/70), and 28.6% (20/70), respectively. Using C-TIRADS 4A as the cutoff, the diagnostic performance after the adjustment based on CEUS results was significantly improved compared with that before the adjustment, with significant different AUCs (0.924 vs. 0.742, *P*=0.003).

## 4. Discussion

Ultrasound examination is the most commonly used method for thyroid nodule examination [3]. Although only 7–15% of thyroid nodules are thyroid cancer [4], overlapping occurs between the conventional ultrasound imaging features of benign and malignant thyroid nodules. For nodules with imaging signs suggesting malignancy, patients often choose surgical treatment due to mental stress. A questionnaire-based survey by the C-TIRADS Special Committee showed that excessive surgery for benign thyroid nodules exists in China [3]. The C-TIRADS guidelines recommend that some nodules such as mummified thyroid nodules can be examined by CEUS to have a comprehensive diagnostic judgment by combining the medical history of the patients with the morphological features of the nodules. In this study, C-TIRADS 4 nodules with a 2–90% risk of malignancy in the thyroid were investigated. The C-TIRADS classification of thyroid nodules was adjusted based on the CEUS results to explore the value of applying CEUS examination in adjusting the categories of C-TIRADS 4 nodules.

In this study, 91.1% (61/67) of malignant thyroid nodules were PTCs based on the pathological examinations. Among them, 47.5% (29/61) were PTMCs, which is consistent with the results reported in a previous study [5]. Numerous studies have shown that PTC is mainly characterized by hypoenhancement and early washout in CEUS, while benign thyroid nodules are mainly characterized by isoenhancement, hyperenhancement, or no enhancement [6–8]. Thus, according to the benign and malignant features of thyroid nodules in CEUS, the C-TIRADS classifications of the thyroid nodules in this study were upgraded or downgraded. Our results showed that the diagnostic performance for C-TIRADS 4 nodules was significantly improved after the adjustment based on CEUS results, with the specificity increasing from 63.8% to 82.8%. In this study, 13 thyroid nodules had their conventional ultrasound imaging showing malignant features, such as solid nodules, suspicious microcalcification, and blurred margins. Among them, 3 nodules were C-TIRADS 4A, and 10 nodules were above C-TIRADS 4A. The CEUS results indicated no enhancement in all 13 of these nodules, and the postoperative histopathological results showed nodular goiter with hemorrhage, cholesterol crystal deposition, and fibrous hyperplasia. Studies have shown that the hemorrhaging cystic degeneration of some benign lesions can be absorbed over time, resulting in shrinkage of the nodules. Conventional ultrasound imaging of such lesions shows similar features to PTC, such as blurred margin and strong internal spot echoes, so the lesions are difficult to distinguish from malignant thyroid nodules. In contrast, CEUS has a high specificity for the diagnosis of such nodules and reduces unnecessary FNA and excessive surgery for benign nodules in the thyroid [3, 8, 9].

In this study, CEUS examination of two nodules showed hyperenhancement, with the histopathological diagnosis of follicular carcinoma. The nodules were classified as C-TIRADS 4A, which were adjusted to one level lower after CEUS found the nodules to be benign. A study reported that ultrasound imaging of this pathological type may misdiagnose nodules as follicular adenoma [10]. However, careful observation of the capsule after the enhancement of the aforementioned nodules showed intermittent blur. Thus, this type of nodule is recommended to be followed up in the clinic. In our study, CEUS results of some PTMC nodules showed benign signs, and the tumor foci under pathological microscopy were small (with the smallest being only 5 mm). Another study [11] showed that the biological behaviors of most PTMCs are mild, and the patients may not show clinical manifestation throughout their lives. The C-TIRADS guidelines indicate in the treatment recommendations section that some maximum diameter 15 mm 4A and maximum diameter 10 mm 4B, 4C nodules not adjacent to the trachea, thyroid envelope, and recurrent laryngeal nerve, and without evidence of lymph nodes and systemic metastasis, namely, the group I nodules in this study, can be follow-up and actively monitored. The present study also showed that the diagnostic accuracy of C-TIRADS 4 nodules after adjustment based on the CEUS results was improved, which would help physicians to more accurately assess the risk of nodule malignancies and choose a reasonable treatment plan. In the present study, although the diagnostic efficiency was improved in both groups, there was statistical significance only in Group II patients, who were with criteria for FNA according to the C-TIRADS guidelines. These results may indicate that CEUS is more necessary for thyroid nodules after C-TIRADS classification by conventional ultrasound, and it minimizes unnecessary FNA and excessive surgery of benign thyroid nodules, thereby conserving medical resources and reducing the anxiety of patients.

This study has some limitations. This study is a single-center study with a small sample size, and it is a retrospective study, so it may have biases. Further multicenter research with large sample sizes is necessary to verify the accuracy of the conclusion of this study.

In summary, the application of CEUS features to adjust the classification of C-TIRADS 4 nodules improved the diagnostic efficiency, especially for thyroid nodules recommended for FNA by the C-TIRADS guidelines. CEUS may be an effective supplement to conventional ultrasound examination of thyroid nodules.

## Figures and Tables

**Table 1:** The diagnostic efficiency of the C-TIRADS 4 before and after the adjustment based on CEUS.

Nodules	Total before the adjustment	Total after the adjustment	Group I before the adjustment	Group I after the adjustment	Group II before the adjustment	Group II after the adjustment
*n*	125	125	55	55	70	70
Sensitivity (%)	83.6(56/67)	91.0(61/67)	84.6(22/26)	84.6(22/26)	82.9(34/41)	95.1(39/41)
Specificity (%)	63.8(37/58)	82.8(48/58)	62.1(18/29)	75.9(22/29)	65.5(19/29)	89.7(26/29)
Accuracy (%)	74.4(93/125)	87.2(109/125)	72.7(40/55)	80.0(44/55)	75.7(53/70)	92.9(65/70)
PPV (%)	72.7(56/77)	85.9(61/71)	66.7(22/33)	75.9(22/29)	77.3(34/44)	92.9(39/42)
NPV (%)	77.1(37/48)	88.9(48/54)	81.8(18/22)	84.6(22/26)	73.1(19/26)	92.9(26/28)
AUC	0.737	0.869	0.733	0.802	0.742	0.924
*Z*	3.288		1.394		2.931	
*P*	0.001		0.163		0.003	

C-TIRADS: the Chinese-Thyroid Imaging Report and Data System; CEUS: contrast-enhanced ultrasound; PPV: positive predictive value; NPV: negative predictive value; AUC: area under the ROC curve.

## Data Availability

The data used to support the findings of this study are available from the corresponding author upon request.
